# Fast and Non-Toxic *In Situ* Hybridization without Blocking of Repetitive Sequences

**DOI:** 10.1371/journal.pone.0040675

**Published:** 2012-07-24

**Authors:** Steen H. Matthiesen, Charles M. Hansen

**Affiliations:** 1 Research and Development, Dako, Glostrup, Denmark; 2 Charles M. Hansen Consulting, Hørsholm, Denmark; University of Campinas, Brazil

## Abstract

Formamide is the preferred solvent to lower the melting point and annealing temperature of nucleic acid strands in *in situ* hybridization (ISH). A key benefit of formamide is better preservation of morphology due to a lower incubation temperature. However, in fluorescence *in situ* hybridization (FISH), against unique DNA targets in tissue sections, an overnight hybridization is required to obtain sufficient signal intensity. Here, we identified alternative solvents and developed a new hybridization buffer that reduces the required hybridization time to one hour (IQFISH method). Remarkably, denaturation and blocking against repetitive DNA sequences to prevent non-specific binding is not required. Furthermore, the new hybridization buffer is less hazardous than formamide containing buffers. The results demonstrate a significant increased hybridization rate at a lowered denaturation and hybridization temperature for both DNA and PNA (peptide nucleic acid) probes. We anticipate that these formamide substituting solvents will become the foundation for changes in the understanding and performance of denaturation and hybridization of nucleic acids. For example, the process time for tissue-based ISH for gene aberration tests in cancer diagnostics can be reduced from days to a few hours. Furthermore, the understanding of the interactions and duplex formation of nucleic acid strands may benefit from the properties of these solvents.

## Introduction

For the past 30 years, formamide has been the solvent of choice in *in situ* hybridization (ISH) for lowering the melting point by destabilizing the double-stranded structure of the nucleic acid helix [1]–[6]. The toxicity of formamide is well known [7], but has been outweighed by its beneficial effects. When hybridizing DNA probes to single locus or low copy number targets on formalin-fixed, paraffin-embedded tissue (FFPE) sections, an incubation of 16 hours or longer is required [6], [8]–[12], which is the major time consuming step in the fluorescence *in situ* hybridization (FISH) procedure (Figure S1). If entire genomes are hybridized, for example, with comparative genomic hybridization, a hybridization time of 48 to 94 hours is often used [13].

Herein, we describe a novel hybridization buffer that dramatically reduces the hybridization time. A buffer that challenges the dogmas of heat-induced denaturation of double-stranded nucleic acids and of blocking against repetitive sequences in probes of genomic origin [14] to perform hybridization. These findings will have a major impact on hybridization based cancer diagnostics and research.

## Materials and Methods

### Specimens

Breast carcinoma, tonsil and colon tissue were obtained from Department of Pathology, Odense University Hospital, Region South, Denmark, fixed in formaldehyde prior to embedding for 24 hours, 24 hours, and 72 hours, respectively. The FFPE tissue blocks were treated as described in the manufacturer’s protocol (Histology FISH Accessory Kit, K5599, Dako, Glostrup, Denmark) and cut in 4 µm sections. Whole blood for making metaphases was obtained from Blodbanken, Glostrup Hospital, Capital Region, Denmark. All specimens and blood were completely anonymized prior to receipt at Dako. According to the Danish law on the Research Ethics Committee System and handling of biomedical research projects and communication between Dako and the Danish Committee on Biomedical Research Ethics and the Regional Ethics Committee (IRB) the tests performed at Dako on anonymous residual tissue and anonymized blood are not subject to an approval by the IRB system because such studies are considered quality control projects. Therefore, no IRB approval for this work has been obtained.

**Figure 1:**
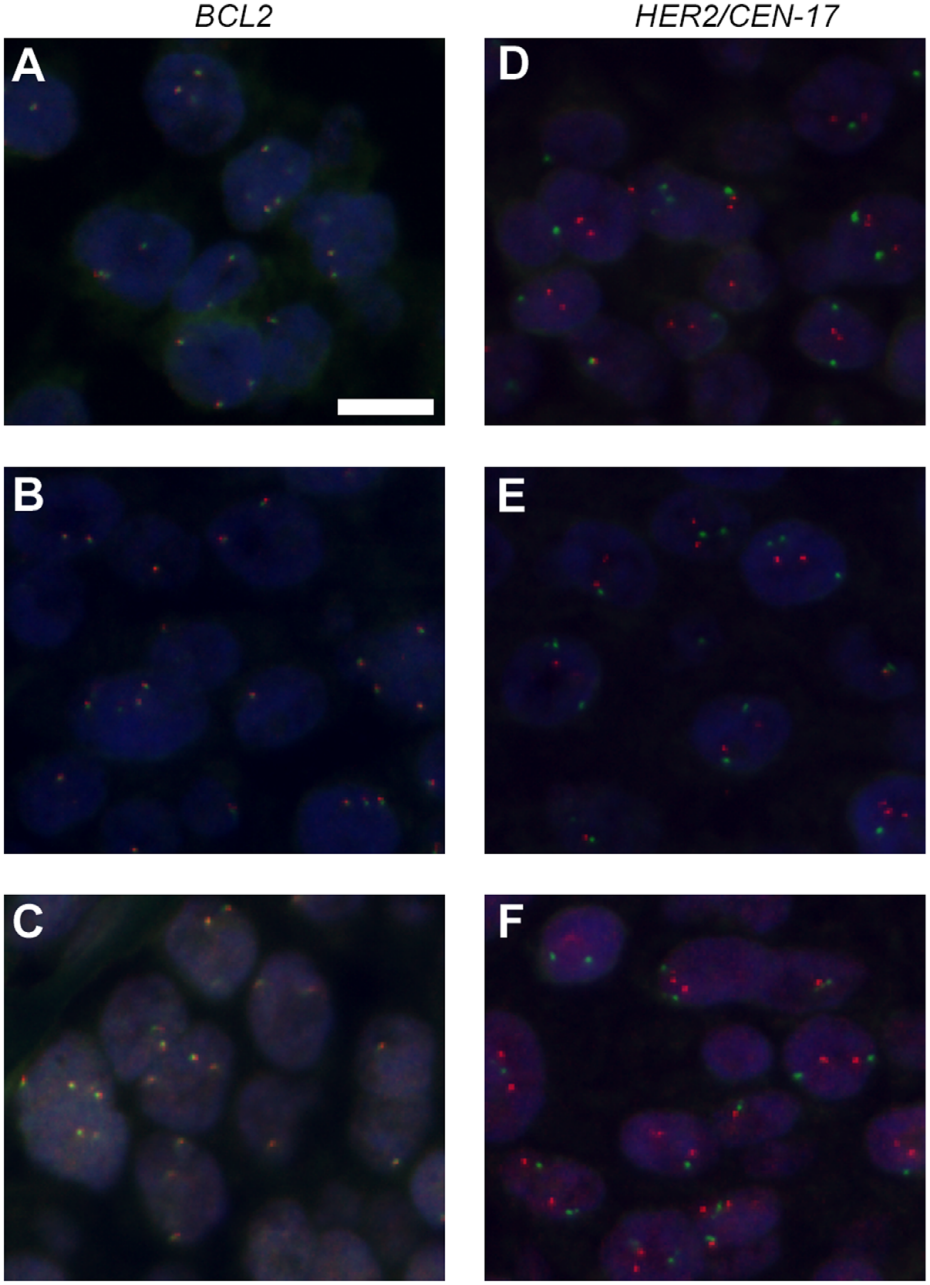
Examples of alternative solvents for FISH hybridization buffer. **A:** sulfolane. **B:** γ-butyrolactone. **C:** Ethylene carbonate (EC). **D:** 2-pyrrolidone. **E:** δ-valerolactam. **F:** EC. The images in **A-C** are merged micrographs of Texas Red-labeled *BCL2* DNA and green fluorescein-labeled *BCL2* DNA split probes, and in **D-F** are of Texas Red-labeled *HER2* DNA and green fluorescein-labeled CEN-17 PNA solid tumor FISH probes on FFPE breast carcinoma tissue sections. The sections were denatured at 67°C for 10 minutes and hybridized at 45°C for 60 minutes with FISH buffers containing 15% solvent. Cot-1 blocking was omitted. All images are taken with same exposure times. Blue, DAPI stain. Scale bar, 10 µm.

### FISH Probes and Buffer

The concentration of Texas Red (TxRed) labeled *HER2* DNA probe (size 218 kb) was 3.3 ng/µL and of fluorescein labeled CEN-17 peptide nucleic acid (PNA) oligo probe, 250 nM (Dako) [15]. The concentration of 20×*HER2* DNA used in Figure S7A + S7B was 67.1 ng/µL. The TxRed labeled *BCL2* DNA probe (size 375 kb, telomeric to the major breakpoint region) was 7.3 ng/µL and fluorescein labeled *BCL2* DNA probe (size 641 kb, centromeric to the major breakpoint cluster region) was 12.9 µg/µL (Dako) [16].

The solvents: ethylene carbonate (E26258), sulfolane (T22209), propylene carbonate ((540013), γ-butyrolactone (B103608), 2-pyrrolidone (240338) and δ-valerolactam (V209) were from Sigma-Aldrich (Copenhagen, Denmark). Formamide was from Invitrogen, Nærum, Denmark (15515-026).

The 15% solvent and formamide buffers consisted of: 15% v/v solvent or formamide; 20% v/v dextran sulfate (D8906, Sigma-Aldrich); 600 mM NaCl; 10 mM citrate buffer; pH 6.2.

The 45% formamide and solvent buffer consisted of: 45% v/v formamide or solvent; 10% v/v dextran sulfate; 0.1 µg/µL Human Cot-1 (15279-011, Invitrogen); 300 mM NaCl; 5 mM phosphate buffer; pH 7.5. The 45% formamide 20×DNA buffer had no Cot-1 added in Figure S7a.

### FISH Procedure

The reagents and protocol used were from Histology FISH Accessory Kit (K5599, Dako) and Cytology FISH Accessory Kit (K5499, Dako). The kits contain all the key reagents, except for probe, required to complete a FISH procedure on FFPE tissue section or cytological specimens. The Dako Hybridizer instrument (S2451) was used for the digestion, denaturation and hybridization steps. The ramping times from the denaturation to hybridization temperature (t = 0) were from 67°C to 45°C in 137±0.60 seconds and from 82°C to 45°C in 197.7±3.2 seconds (mean ± S.D., n  = 3) at an ambient temperature of 21°C.

The FFPE sections from human tissues (tonsil, breast carcinoma and colon) were baked at 60°C for 60 minutes, deparaffinated in xylene baths, rehydrated in ethanol baths and then transferred to Wash Buffer. The samples were then pre-treated in Pre-Treatment Solution at a minimum of 95°C for 10 minutes using a microwave oven (JT356, Whirlpool Nordic, Herlev, Denmark), washed 2 × 3 minutes, digested with Pepsin at 37°C for 3 minutes, washed 2 × 3 minutes, dehydrated in a series of ethanol baths, and air dried. Next, the samples were incubated with 10 µL FISH probe as described for the individual experiments, washed in Stringency Buffer at 65°C 10 minutes, washed 2 × 3 minutes, dehydrated in a series of ethanol baths, and air dried. Finally, the slides were mounted with 15 µL Fluorescence Mounting Medium.

Metaphase preparations of normal blood cells were fixed in 3.7% formaldehyde for 2 minutes and washed 2 × 5 minutes. The samples were dehydrated in a series of ethanol baths and air dried. The samples were then incubated with 10 µL FISH probe as described in the legend of Figure S4, washed in Stringency Buffer at 65°C 10 minutes, washed 2 × 3 minutes. After wash, the samples were dehydrated in a series of ethanol baths, and air dried. Finally, the slides were mounted using 15 µL Fluorescence Mounting Medium.

### Fluorescence Microscopy and Image Analysis

Fluorescence imaging was performed using a Leica DM6000B microscope with a HCX PL APO 40×/1.25-0.75 Oil objective (Leica Microsystem Imaging System, Leica Microsystems A/S, Ballerup, Denmark) and a 100 W mercury lamp (Leica). Images were acquired in grayscale using a DFC300 FX R2 digital CCD camera (Leica) using Leica QFISH acquisition software.

For the image analysis performed, ImageJ, v 1.45a (http://rsbweb.nih.gov/ij/) software was used. High intensity pixels were identified by adjusting the brightness/contrast values, which did not affect the quantification. The total intensity in the region of interest (ROI) that surrounded each signal spot was measured. The ROI size was set to three times the width of the point spread function (3 × 3 pixels) to correct for local background [17]. The individual spots and a nuclei background region (mean of 8 × 8 pixels) were quantified on the original image, and subsequently the background was subtracted. The analysis was performed on three images of each specimen, and 15 signals from each were quantified giving a total of 45 signals for each time point and color. Manual evaluation of the FISH staining was performed with the same Leica DM6000B fluorescence microscope as for imaging using DAPI, FITC, TxRed single filters and a FITC/TxRed double filter, a 10×ocular and 10×, 20×, 40×, and 100×oil objectives.

**Figure 2:**
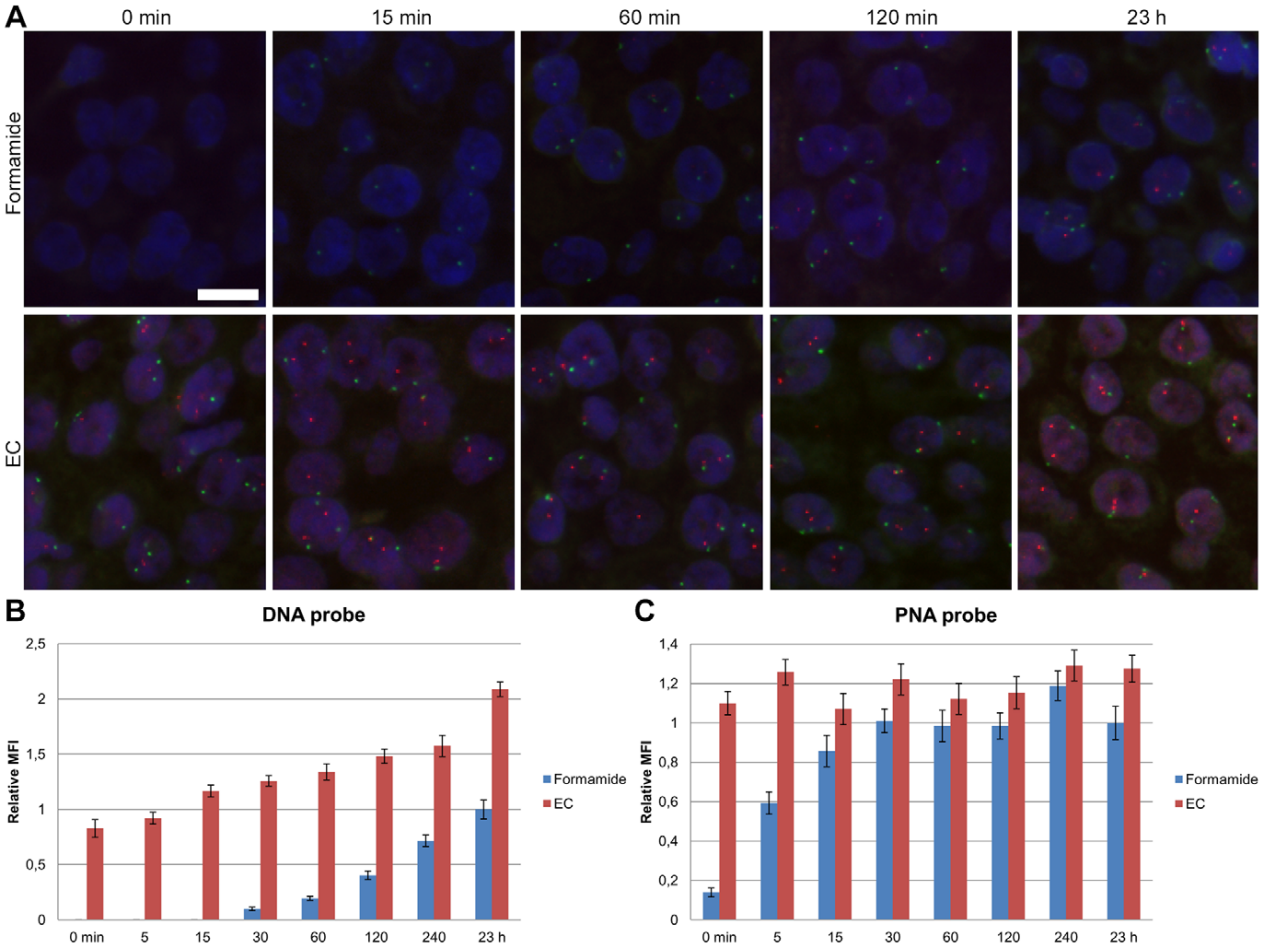
Effect of EC on hybridization rate. Time chase comparison of FISH signal intensities using 15% EC buffer and 45% formamide buffer on FFPE breast carcinoma tissue sections. Cot-1 blocking was used in formamide buffer, but omitted for EC buffer. Identical probe concentration was used in the two buffers. EC buffer was denatured at 67°C for 10 minutes, formamide buffer at 82°C for 5 minutes, and then both hybridized at 45°C from t = 0 minute to t = 23 hours. **A:** Merged micrograph of red *HER2* DNA probe signals, green CEN-17 PNA signals and blue DAPI staining (Figure S2). The images are taken with identical exposure times. Scale bar, 10 µm. **B:** Quantitative analysis of *HER2* DNA and **C,** of CEN-17 PNA signals (Figures S2 and S3 **)**. MFI, mean fluorescence intensity. The bars represent the 95% confidence interval (n = 45 signals).

## Results

### New FISH Hybridization Buffer

In the search for formamide substitutes that are less toxic and can reduce the hybridization time, the Hansen solubility parameters for DNA [18] were used as a guidance to identify potential replacement candidates. From screening potential candidates, a pattern emerged showing that the solvents capable of supporting fast hybridization of 1 hour were highly polar aprotic with a relatively low hydrogen-bonding solubility parameter (δ_H_; below 13 MPa^1/2^) and a molar volume below 110 cm^3^/mole (Table S1). The new category of solvents is characterized by lower miscibility with hybridization buffer components than formamide. Therefore, when the buffer components were used in the same concentrations as in a traditional 45% formamide FISH buffer, the hybridization buffers separated into two phases. Using these two-phase buffers, a shorter hybridization time was required than with formamide; however, the FISH stains showed both higher background and heterogeneous signal intensities. A buffer consisting of 15% v/v solvent, 20% v/v dextran sulfate, 600 mM NaCl and 10 mM citrate buffer pH 6.2, was developed to hinder phase separation and to decrease background staining. The buffer composition thereby contained one third of the solvent and double the concentration of both dextran sulfate and NaCl than the traditional formamide FISH buffer [14], [19], [20]. Using lower solvent concentration than 15% v/v reduced the staining intensities. In Figure 1, FISH stainings using five of the identified fast hybridization solvents in the new buffer composition are shown. One of the solvents - ethylene carbonate (EC) - was the only tested solvent buffer that stayed in one phase at room temperature. The EC buffer also showed major advantages compared to a formamide buffer. Firstly, it resulted in an increased hybridization rate and did not require blocking against repetitive sequences [14]. Secondly, by lowering the denaturation temperature from 82°C to 67°C, background staining was reduced. Last, but not least, EC is non-toxic at the concentration used [7], [21]–[23].

### Time-course Testing

A time-course experiment was performed on FFPE breast carcinoma tissue to examine the signal intensities obtained using EC and formamide buffers at different hybridization time points (Figure 2 and Figure S2). The fluorescent signals from the *HER2* (*ERBB2*; *NEU* or *CD340*) DNA probe, an important marker in breast and gastric cancer diagnostics [8], [9], [24], could already be observed at t = 0 minutes in EC buffer. This indicate that the 137 seconds it took to cool specimens down from denaturation at 67°C to 45°C, where hybridization was instantly stopped in stringent wash buffer, was sufficient to allow hybridization that generated visible signals from the DNA probe. Using image analysis, the quantified mean fluorescence intensity (MFI) showed significantly higher values at all time points for the *HER2* DNA probe in EC buffer with extremely low *P* values (*P*<0.0001, t-test two sample means with equal variance, n_1_ = 45 and n_2_ = 45). When compared to the formamide buffer after 23 h, the MFI showed equivalent DNA probe signal intensities after 5 minutes hybridization (*P*<0.13) in EC buffer and significantly higher values after 15 minutes (*P*<0.0018) using the EC buffer. This is approximately 100 times shorter hybridization time in EC buffer compared to the traditional formamide buffer. The quantification data corresponded to the manual scoring of signal intensities (Figure S3). The chromosome centromere 17 (CEN-17) peptide nucleic acid (PNA) probe, which is a reference probe used in HER2 testing, also showed faster hybridization kinetics in the EC buffer. The signals using EC buffer showed maximum intensity at t = 0 minute hybridization, and equivalent intensity in formamide buffer was first obtained when a plateau was reached at t = 30 minutes (Figure 2C). At all time points from 0–30 minutes, the MFI was significant higher with extremely low *P* values (*P*<0.0001, t-test two sample means with equal variance, n_1_ = 45 and n_2_ = 45) for the CEN17 PNA probes in EC buffer compared to formamide buffer. PNA is known to hybridize fast due to the uncharged nature of its backbone [25], and 15–30 minutes corresponds to the normal time required to obtain maximum signal intensities for PNA probes using formamide buffer. The background staining for the DNA probe in the EC buffer increased when the hybridization time reached 240 minutes (Figure S2). The optimal signal-to-noise ratio was at 60–120 minutes hybridization. Beyond 120 minutes, the signal-to-noise ratio decreased as the signal intensities leveled off, while background further increased (Figure S2). The evaporation of primarily water and the breakdown of EC to ethylene glycol and CO_2_, which is catalyzed by salt and heat, may be contributing factors for the increased background over time.

**Figure 3:**
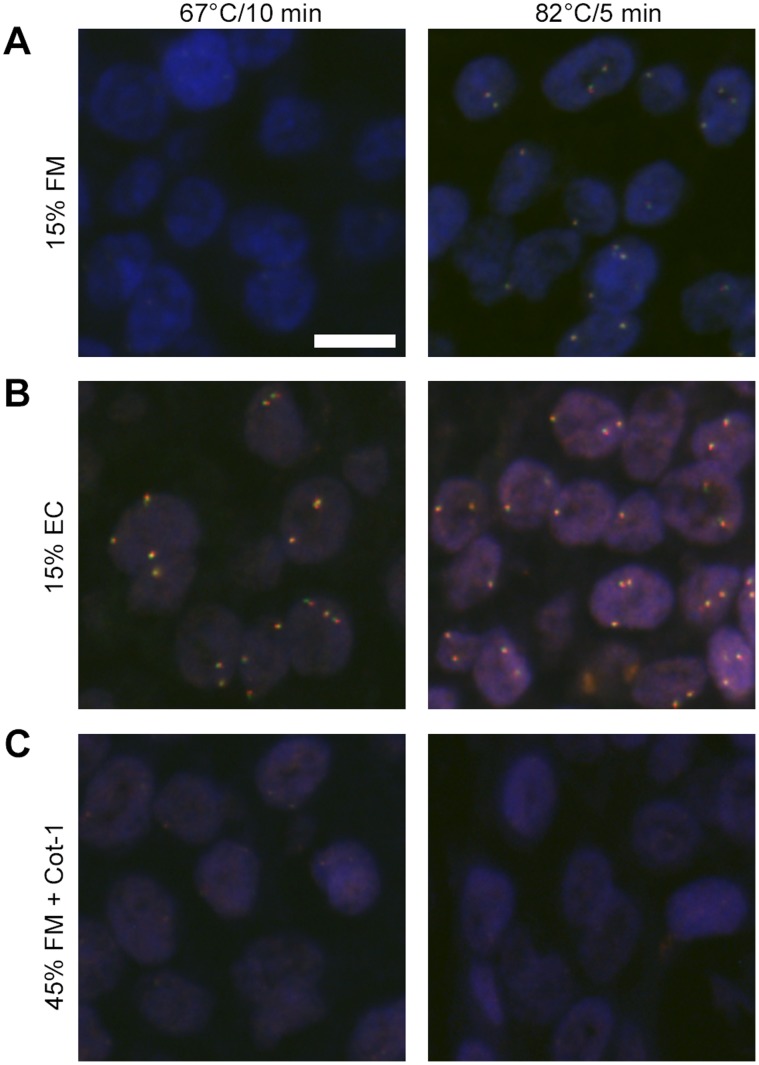
FISH stains using same buffer component concentrations for EC and formamide buffers. *BCL2* in: **A:** 15% formamide buffer, **B:** 15% EC buffer and **C:** 45% formamide buffer with Cot-1 blocking, with different denaturation conditions on FFPE breast carcinoma tissue sections. Merge of green *BCL2* DNA, red *BCL2* DNA split probe signals and of blue DAPI staining. Denatured at 67°C for 10 minutes or 82°C for 5 minutes and hybridized at 45°C for 60 minutes. The images are taken with identical exposure times. Scale bar, 10 µm.

**Figure 4:**
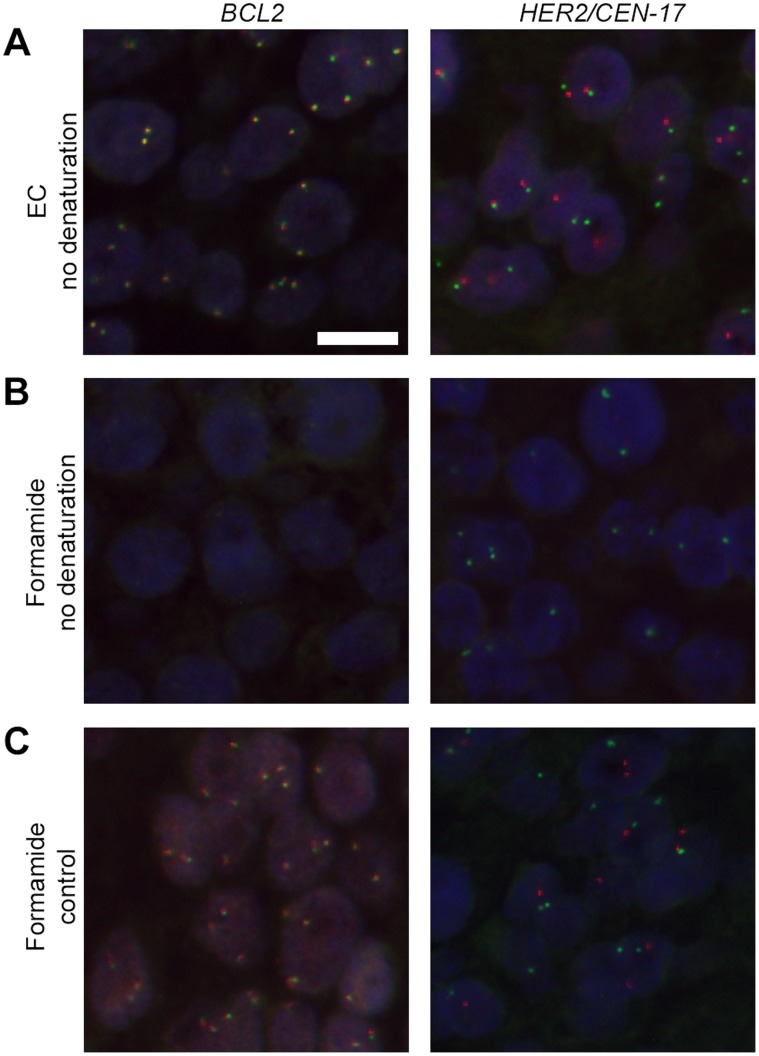
FISH stains without denaturation. **A** and **B:** No denaturation and hybridization in 15% EC buffer or 45% formamide buffer at 50°C for 120 minutes. **C:** The control was denatured using 45% formamide buffer at 82°C for 5 minutes and hybridized at 45°C for 16 hours. The images in **A-C**, are merged micrographs of red *BCL2* DNA and green *BCL2* DNA split probes, and red *HER2* DNA and green CEN-17 PNA probes on FFPE breast carcinoma tissue sections. The images are taken with identical exposure times. Blue, DAPI stain. Scale bar, 10 µm.

### Multi-Specimens

To examine if the fast hybridization was a specimen phenomenon, different types of specimens were tested (Figure S4). Background staining with EC buffer was more pronounced in FFPE breast tissue than in FFPE tonsil, colon, kidney and gastric tissues. This is probably correlated to the higher levels of connective tissue and fat cells in breast tissue. Besides FFPE tissue, we tested metaphase preparations using a typical lymphoma translocation marker [10], [12], *BCL2* FISH split probe, and showed that the signal intensity at 60 minutes hybridization was considerably higher using the EC buffer compared with the traditional formamide buffer (Figure S5). No R-bands on metaphases were observed despite omitting Cot-1 [14], [15] blocking against repetitive sequences. The specific chromosome location of e.g. the BCL2 probe showed high probe specificity. Testing other probes including *ALK*, *CCND1*, CEN-7, *IGH*, *IGK, c-MYC*, *TCRAD, HER2* and *TOP2A* showed no sign of changes in probe specificity (data not shown). This suggests that the specificity of DNA probes and PNA oligos is conserved in the EC buffer. Thus, faster kinetics and specific binding were observed with different FFPE tissue specimens and with cytological metaphase spreads.

### Buffer Composition

To investigate if the increased hybridization rate was caused by the change in the relative buffer composition, i.e. the percentage of solvent, dextran sulfate and salt, a 15% v/v formamide buffer was compared with the equivalent 15% v/v EC buffer (Figure 3). This showed a minor improvement in signal intensities at t = 60 minutes with high denaturation temperature (82°C) and suggests that a 15% v/v formamide buffer composition increases the hybridization kinetics compared with the traditional formamide buffer. However, the signal intensities using the EC buffer were still considerably stronger. The increased signal intensities observed at lower formamide concentration supports the notion that the traditional formamide concentration hinders fast hybridization kinetics [26] and that high salt concentrations facilitates fast hybridization kinetics. There was no indication of lack of *BCL2* or *HER2* probe specificity.

### No Denaturation

Next, we examined if classic heat-denaturation was required to obtain efficient hybridization with the EC buffer. The EC hybridization buffer was indeed able to generate strong signals and low background in the absence of heat-denaturation of both the probe and target prior to hybridization, when hybridized at 45°C overnight (Figure S6). Increasing the hybridization temperature to 50°C and only 120 minutes hybridization resulted in strong signal intensities for the DNA probes using the EC buffer, whereas no signals were obtained using formamide buffer at the same settings (Figure 4).

### Increased Probe Concentration

After denaturation, the complementary strands are brought together under conditions that favor hybridization. This can be achieved by creating an environment with high probe concentration at a lowered temperature. In classic formamide buffers, the dextran sulfate is believed to raise the effective probe concentration and thereby increase the kinetics [4], [27], [28]. To examine if the faster hybridization rate was due to a higher effective concentration of the DNA probe in the EC buffer, a 20×DNA probe concentration was used with the formamide buffer (Figure S7). An increase in signal intensity was observed using formamide buffer, but still not at the same level as 1×DNA probe concentration in EC buffer. The background staining was excessive when the formamide buffer was not blocked with 1×Cot-1, compared with the EC buffer. Neither increasing the denaturation temperature to 82°C for 5 minutes nor increasing the Cot-1 concentration 20 times improved the results with the formamide buffer (data not shown).

## Discussion

The solvents identified in this paper, for example EC, do not compete as hydrogen donors for base pairing to the same extent as formamide (δ_H_ parameter, Table S1; Discussion S1; Table S2). This suggests that the solvents have a lower affinity for attachment to the bases through hydrogen bonds. This lower affinity towards base pairing could be a part of the mechanism for faster hybridization of the strands. The strands can gain easier access and bind to their complementary strands when there are no hydrogen bonds from the solvent disturbing the base pairing [5], [26]. An analogy is a zipper that can be opened and closed fast, unless objects, in this case formamide, are stuck in the zipper and thereby hindering the assembly until they are removed. The stability of the DNA helix is primarily caused by hydrophobic stacking and not by base pairing [29], [30]. The base pairing ensures that the bases are closely packed such that stacking can occur. The observed effect of re-annealing without prior heat-induced denaturation or with a low heat denaturation temperature supports the notion that polar aprotic solvent buffers decrease the stability of the helix. We propose that the mechanism of the solvents is not by attacking hydrogen bonds, but instead by diminishing the hydrophobic stacking of bases and thereby decreasing the stability of the helix.

Repetitive sequences re-anneal much faster than unique gene sequences [31]–[33]. A decreased stability of the DNA helix might result in an even faster re-annealing of the repetitive sequences in both the probe and the genome after denaturation. Therefore, we suggest that they re-anneal to themselves, and as a consequence, DNA blocking with Cot-1 can be omitted.

The new hybridization solvents are strong candidates to replace the use of classic formamide as the preferred solvent in molecular biology due to their properties to lower the melting temperature, increase the hybridization rate and decrease health risks. In addition to the results shown in this paper, they also work well for e.g. LNA (Locked Nucleic Acids) and DNA oligo probes, RNA detection, as denaturants and for stringent wash (WO 2010/097655; WO 2010/097656; WO 2010/097707). The shortened hybridization time of the IQISH technology will have a major impact on ISH based cancer diagnostic as the turnaround time from sample to diagnosis makes a difference for the patient.

## Supporting Information

Figure S1 **Timeline for FISH procedure for single locus targets performed on FFPE tissue.**
(PDF)

Figure S2 **Time-chase comparison between formamide buffer and EC buffer.**
(PDF)

Figure S3 **Manual scoring of signal intensities in time-chase experiments using formamide and ethylene carbonate buffers.**
(PDF)

Figure S4 **Examples of FISH on different FFPE tissue sections using EC buffer.**
(PDF)

Figure S5 **FISH on metaphases using EC buffer and formamide buffer.**
(PDF)

Figure S6 **Examples of FISH on different FFPE tissue sections using EC buffer without denaturation.**
(PDF)

Figure S7 **FISH with 20×** ***HER2*** **DNA probe concentration.**
(PDF)

Table S1 Hansen solubility parameter (HSP) – DNA.
(PDF)

Table S2 HSP- Formamide and EC with water.
(PDF)

Discussion S1 HSP considerations in relation to the suggested base stacking mechanism. Please also see Table S1 and S2.
(PDF)
