# Supplementary material for: Fast and Non-Toxic In Situ Hybridization without Blocking of Repetitive Sequences
Source: PLoS One. 2012 Jul 24;7(7):e40675. doi: 10.1371/journal.pone.0040675 (PMC3404051; doi:10.1371/journal.pone.0040675)
Supplement: Figure S1 — Timeline for FISH procedure for single locus targets performed on FFPE tissue. (PDF) [file pone.0040675.s001.pdf]

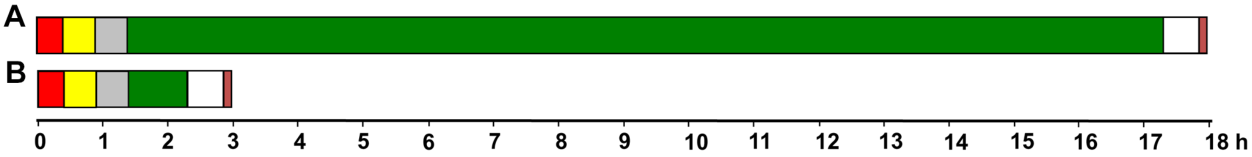


**Figure S1. Timeline for FISH procedure for single locus targets performed on FFPE tissue**. **A:** Traditional formamide buffer with 16 hours hybridization. **B:** Buffer with 1 hour hybridization. Red, deparaffinization; yellow, heat pre-treatment; gray, digestion and denaturation; green, hybridization; white, stringent wash; brown, mounting. See Material and Methods for assay details.
