# Supplementary material for: Fast and Non-Toxic In Situ Hybridization without Blocking of Repetitive Sequences
Source: PLoS One. 2012 Jul 24;7(7):e40675. doi: 10.1371/journal.pone.0040675 (PMC3404051; doi:10.1371/journal.pone.0040675)
Supplement: Figure S2 — Time-chase comparison between formamide buffer and EC buffer. (PDF) [file pone.0040675.s002.pdf]

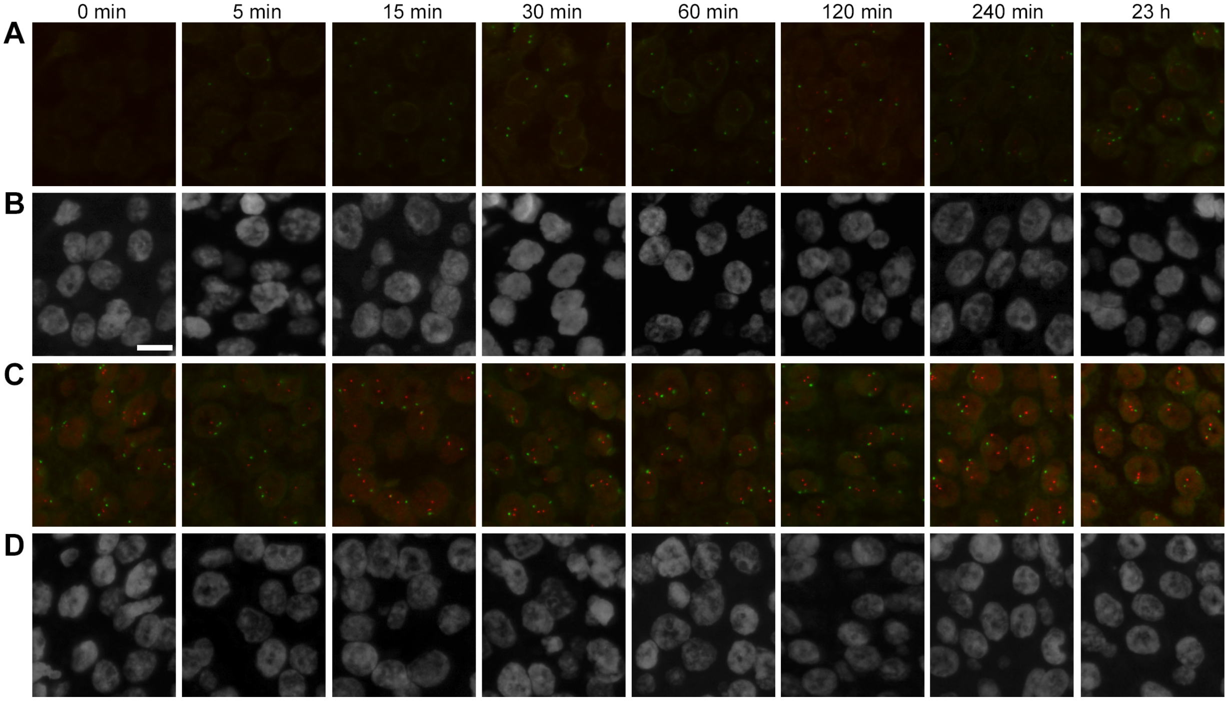

Figure S2. **Time-chase comparison between formamide buffer and EC buffer.** Time-chase comparison of FISH signal intensities using 15% EC buffer and 45% formamide buffer on FFPE breast carcinoma tissue sections. **A:** Forty-five percent formamide buffer with Cot-1 blocking. Denaturation at 82°C for 5 minutes, then hybridized at 45°C from t=0 minute to t=23 hours. **C:** Fifteen percent EC buffer without Cot-1 blocking. Denaturation at 67°C for 10 minutes, then hybridized at 45°C from t=0 minute to t=23 hours. **A** and **C:** Merge of red *HER2* DNA probe signals, green CEN-17 PNA signals. **B** and **D:** DAPI staining of sections in **A** and **C**, respectively. Images are taken with identical exposure times. Scale bar, 10 µm.
