# Supplementary material for: Fast and Non-Toxic In Situ Hybridization without Blocking of Repetitive Sequences
Source: PLoS One. 2012 Jul 24;7(7):e40675. doi: 10.1371/journal.pone.0040675 (PMC3404051; doi:10.1371/journal.pone.0040675)
Supplement: Figure S3 — Manual scoring of signal intensities in time-chase experiments using formamide and ethylene carbonate buffers. (PDF) [file pone.0040675.s003.pdf]

A

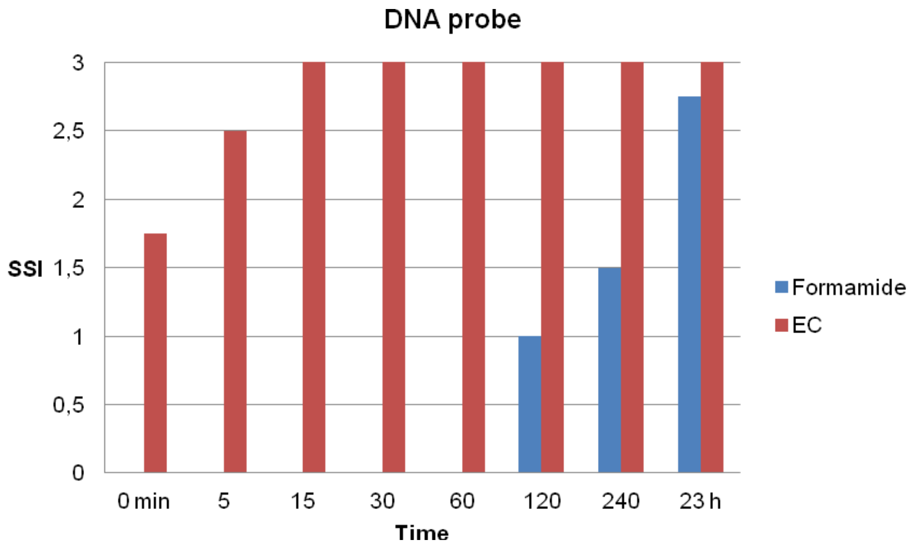

B

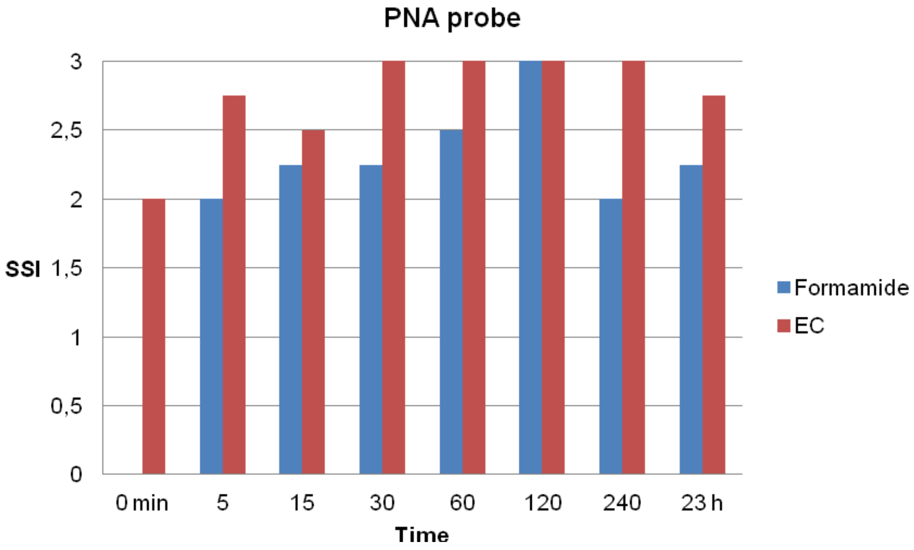

Figure S3. Manual scoring of signal intensities in time-chase experiments using formamide and ethylene carbonate buffers. A: *HER2* DNA probe. **B:** CEN-17 PNA probe. SSI, Scored Signal Intensity. The signal intensities were evaluated on a 0–3 scale. Score: 0, no signal; 1, weak signal intensity; 2, moderate signal intensity; 3, strong signal intensity. Between 0 and 3, additional grades 0.5 apart were used by the observer to assess signal intensity.
