# Supplementary material for: Fast and Non-Toxic In Situ Hybridization without Blocking of Repetitive Sequences
Source: PLoS One. 2012 Jul 24;7(7):e40675. doi: 10.1371/journal.pone.0040675 (PMC3404051; doi:10.1371/journal.pone.0040675)
Supplement: Figure S4 — Examples of FISH on different FFPE tissue sections using EC buffer. (PDF) [file pone.0040675.s004.pdf]

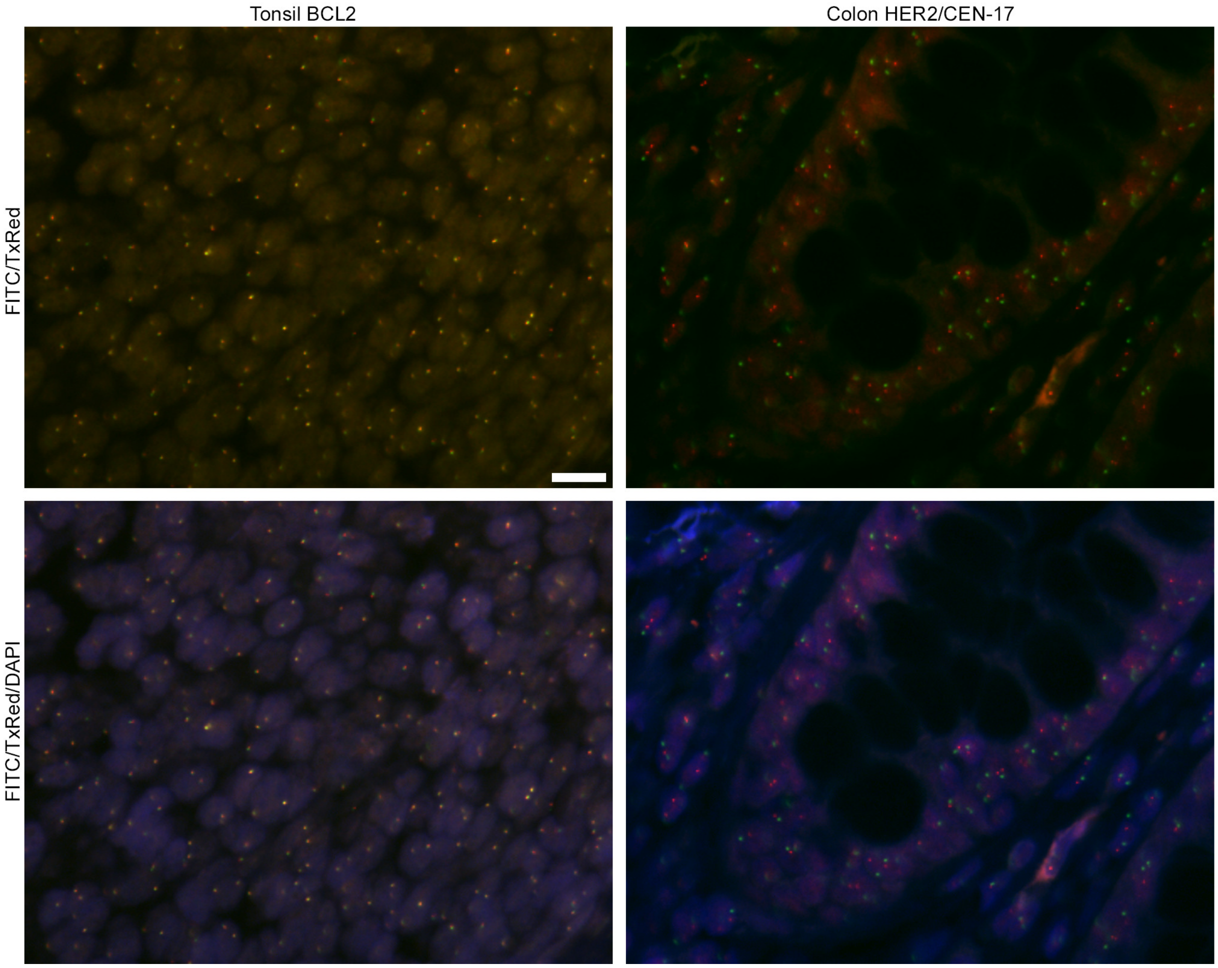


**Figure S4. Examples of FISH on different FFPE tissue sections using EC buffer.** *BCL2* and *HER2*/CEN-17 probe in 15% EC buffer on FFPE tonsil and colon tissue sections. Denaturation at 67°C for 10 minutes and hybridization at 45°C for 60 minutes. No Cot-1 blocking. FITC/TxRed, merge of green and red *BCL2* DNA split probe signals. Merge of red *HER2* DNA probe signals and green CEN-17 PNA signals. DAPI, blue staining. Scale bar, 10 µm.
