# Supplementary material for: Fast and Non-Toxic In Situ Hybridization without Blocking of Repetitive Sequences
Source: PLoS One. 2012 Jul 24;7(7):e40675. doi: 10.1371/journal.pone.0040675 (PMC3404051; doi:10.1371/journal.pone.0040675)
Supplement: Figure S5 — FISH on metaphases using EC buffer and formamide buffer. (PDF) [file pone.0040675.s005.pdf]

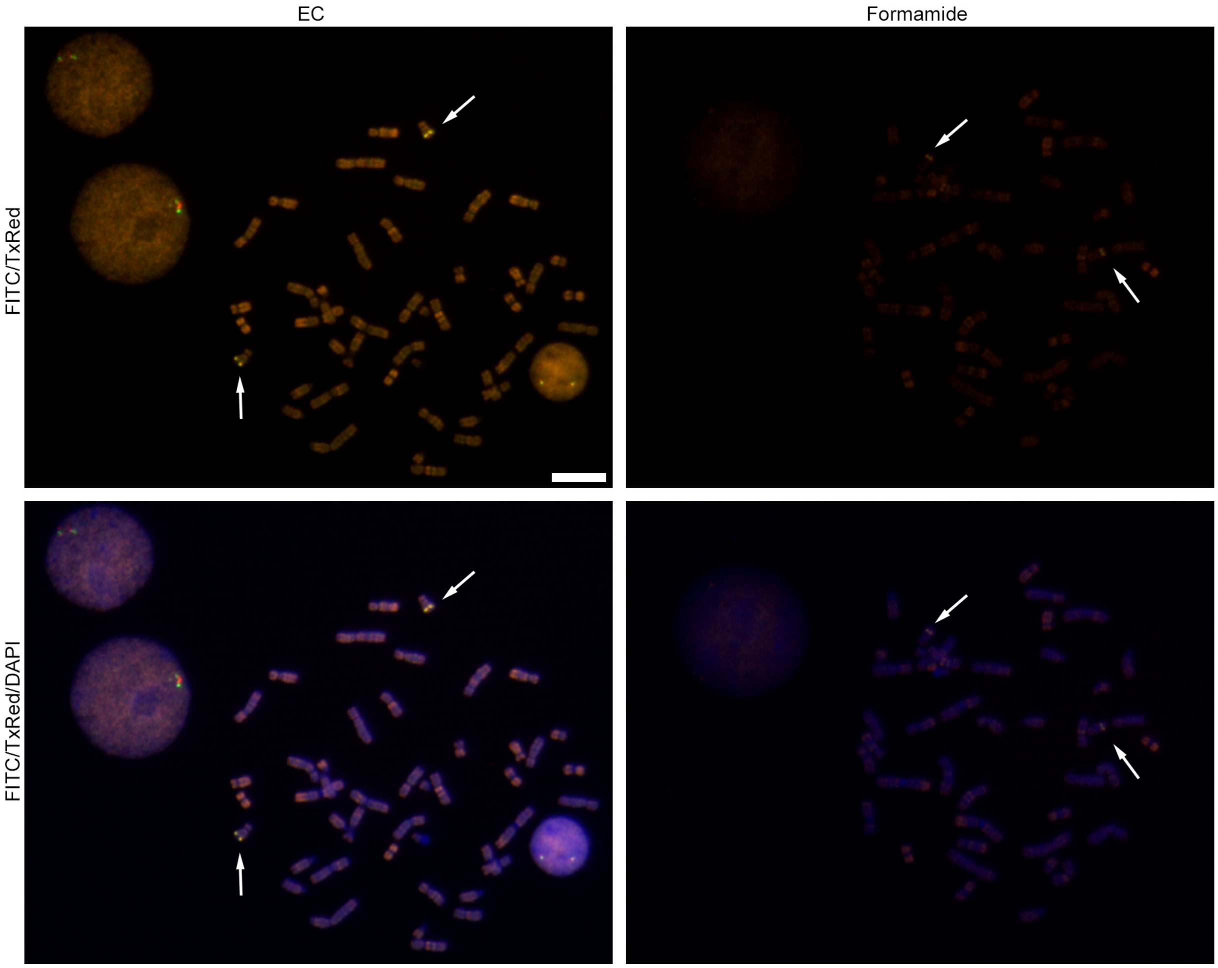

Figure S5. FISH on metaphases using EC buffer and formamide buffer. FISH on metaphase spreads using 15% EC buffer without Cot-1 blocking and 45% formamide buffer with Cot-1 blocking. Denaturation at 82°C for 5 minutes, then hybridization at 45°C for 60 minutes. FITC/TxRed, merge of green and red *BCL2* DNA split probe signals. DAPI, blue staining. Images are taken with identical exposure times. Arrows, show probe location. Scale bar, 10 µm.

.
