# Supplementary material for: Fast and Non-Toxic In Situ Hybridization without Blocking of Repetitive Sequences
Source: PLoS One. 2012 Jul 24;7(7):e40675. doi: 10.1371/journal.pone.0040675 (PMC3404051; doi:10.1371/journal.pone.0040675)
Supplement: Figure S6 — Examples of FISH on different FFPE tissue sections using EC buffer without denaturation. (PDF) [file pone.0040675.s006.pdf]

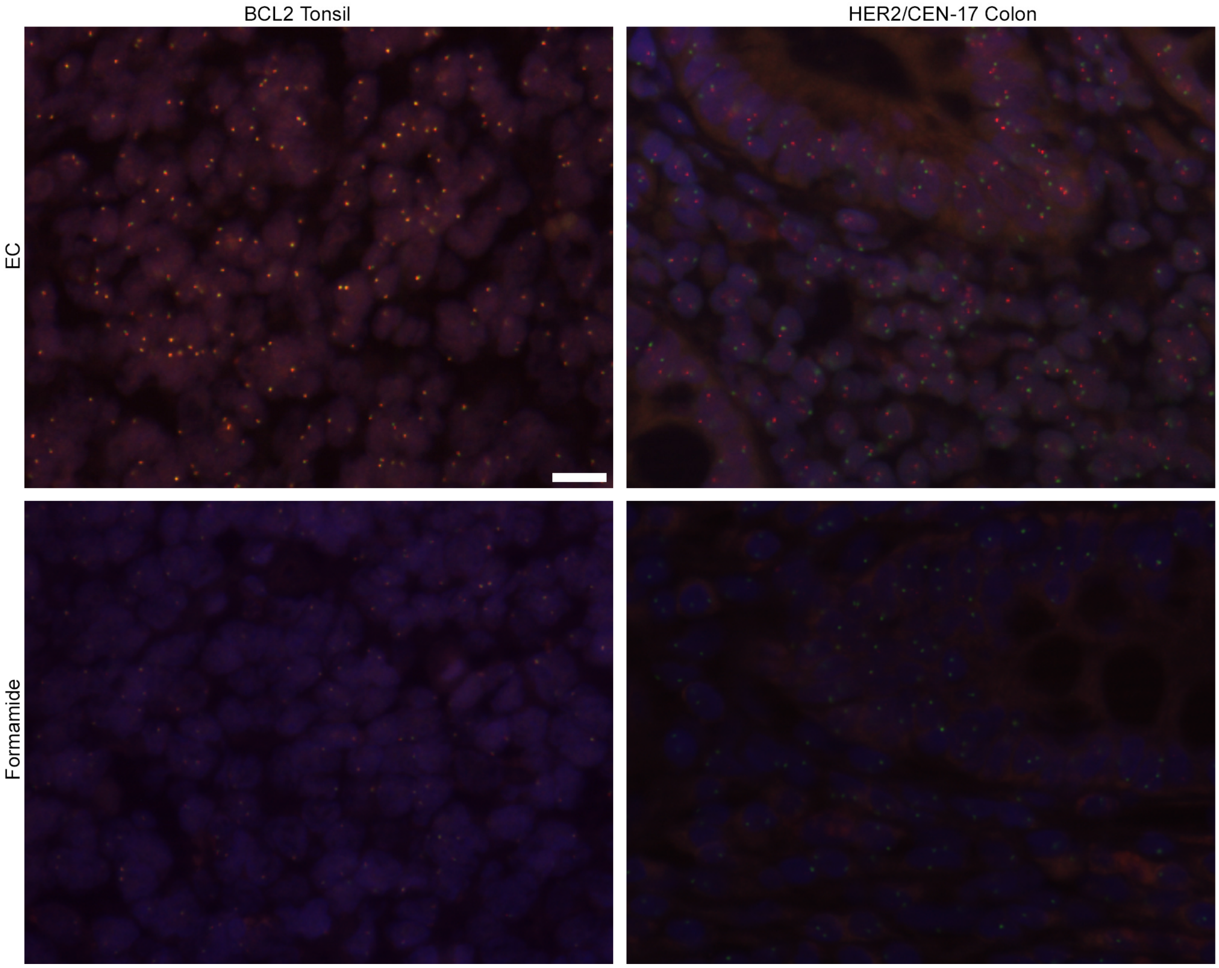

Figure S6. **Examples of FISH on different FFPE tissue sections using EC buffer without denaturation.** The FFPE tissue sections of tonsil and colon were hybridized with 15% EC buffer and 45% formamide buffer at 45°C for 16 hours without prior denaturation. The images are merged micrographs of red *BCL2* DNA and green *BCL2* DNA split probes, and red *HER2* DNA and green CEN-17 PNA probes. The images are taken with identical exposure times. DAPI, blue staining. Scale bar, 10 μm.
