# Supplementary material for: Fast and Non-Toxic In Situ Hybridization without Blocking of Repetitive Sequences
Source: PLoS One. 2012 Jul 24;7(7):e40675. doi: 10.1371/journal.pone.0040675 (PMC3404051; doi:10.1371/journal.pone.0040675)
Supplement: Figure S7 — FISH with 20× HER2 DNA probe concentration. (PDF) [file pone.0040675.s007.pdf]

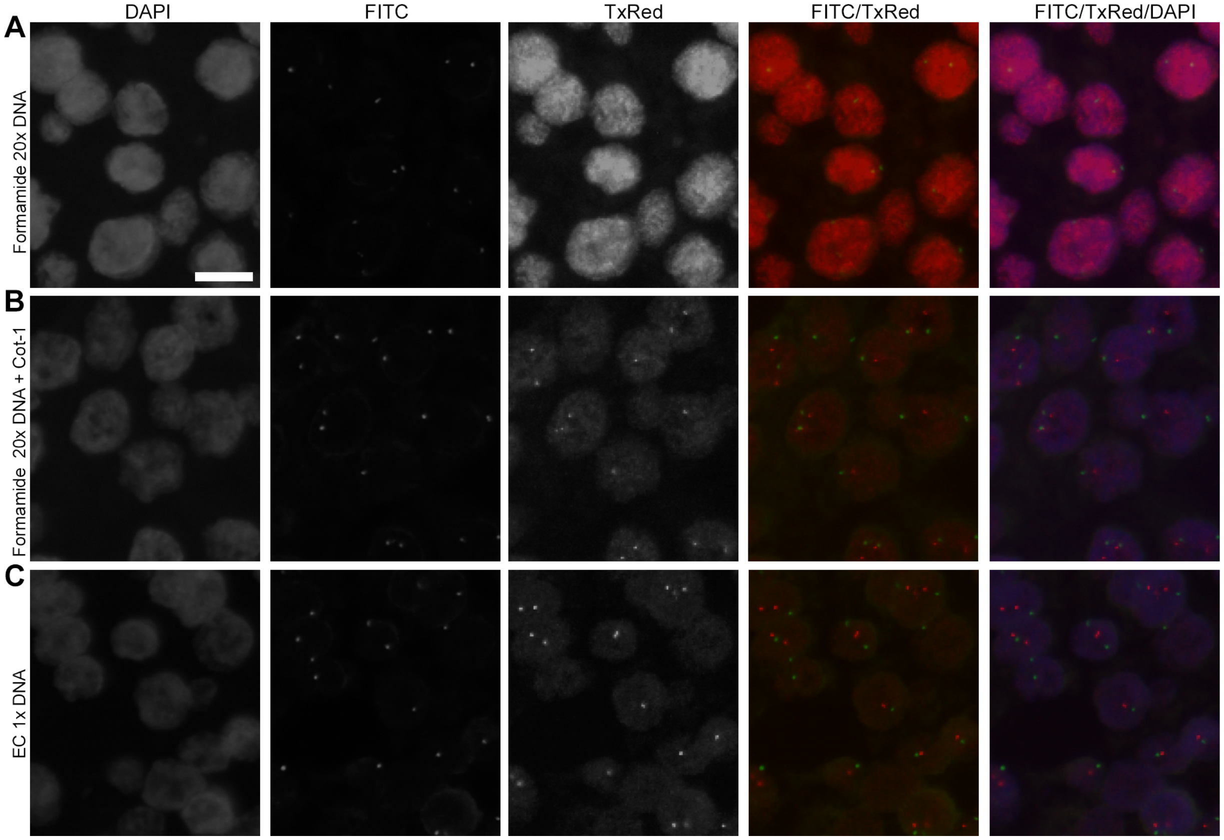

Figure S7. **FISH with 20x *HER2* DNA probe concentration. A:** Forty-five percent formamide buffer with 20x DNA concentration. **B:** Forty-five percent fomamide buffer with 20x DNA concentration and 1x Cot-1. **C:** Fifteen percent EC buffer with 1x DNA concentration. CEN-17 PNA probe concentration was kept constant. The images in FITC/TxRed are merged micrographs of green CEN-17 PNA probes and red *HER2* DNA. The FFPE breast carcinoma tissue sections were denaturated at 67°C for 10 minutes and hybridized at 45°C for 60 minutes. The images are taken with identical exposure times. DAPI, is stained blue in FITC/TxRed/DAPI. Scale bar, 10 µm.
