# Supplementary material for: Fast and Non-Toxic In Situ Hybridization without Blocking of Repetitive Sequences
Source: PLoS One. 2012 Jul 24;7(7):e40675. doi: 10.1371/journal.pone.0040675 (PMC3404051; doi:10.1371/journal.pone.0040675)
Supplement: Table S1 — Hansen solubility parameter (HSP) – DNA. (PDF) [file pone.0040675.s008.pdf]

Table S1. Hansen solubility parameter (HSP) - DNA

Solvents	δ_D	δ_P	δ_H	RED	V (cm^3/mole)
2-pyrrolidone	19.4	17.4	11.3	0.249	76.4
Sulfolane	20.3	18.2	10.9	0.288	95.7
δ-valerolactam	20.0	16.4	8.4	0.443	100.0
γ-butyrolactone	19.0	16.6	7.4	0.450	76.5
EC	19.4	21.7	5.1	0.563	66.0
Propylene carbonate	20.0	18.0	4.1	0.678	85.2
Formamide	17.2	26.2	19.0	0.977	39.8
Urea	20.9	18.7	26.4	1.447	45.8

Examples of HSP for fast acting hybridization ISH solvents are shown in the table. The calculated solubility sphere for DNA is based on experimental data [29] and was used as an aid to identify possible alternatives to formamide. DNA [18]: $\delta_D = 19.0$; $\delta_P = 20.0$; $\delta_H = 11.0$; R_0=11; FIT = 1.000; NO = 12. The HSP are at the center of the sphere. Ro, the radius of the sphere, suggests the maximum difference in affinity tolerable for a "good" interaction, but there can be boundary uncertainty. δ_D, δ_P and δ_H account for the cohesion energy density from atomic dispersion interactions, molecular polar interactions and molecular hydrogen-bonding interactions in $MPa^{½}$. RED (Relative Energy Difference, R_a/R_0) numbers less than 1.0 indicate high affinity; RED numbers equal or close to 1.0 indicate boundary conditions; and progressively higher RED numbers indicate progressively lower affinities. V, molar volume. $R_a^2 = 4(\delta_{D2}-\delta_{D1})^2 + (\delta_{P2}-\delta_{P1})^2 + (\delta_{H2}-\delta_{H1})^2$.
