# Supplementary material for: Fast and Non-Toxic In Situ Hybridization without Blocking of Repetitive Sequences
Source: PLoS One. 2012 Jul 24;7(7):e40675. doi: 10.1371/journal.pone.0040675 (PMC3404051; doi:10.1371/journal.pone.0040675)
Supplement: Table S2 — HSP- Formamide and EC with water. (PDF) [file pone.0040675.s009.pdf]

Table S2. HSP- Formamide and EC with water

	δ_D	δ_P	δ_H	R_0
Formamide	17.2	26.2	19.0	-
Formamide/water 12/88 v/v	18.0	18.2	17.2	-
EC	19.4	21.7	5.1	-
EC/Water 50/50 v/v	18.7	19.3	11.0	-
DNA correlation [18]	19.0	20.0	11.0	11.0
Water miscibility correlation [18]	18.1	17.1	16.9	13.0

The optimum amount of formamide in water with regard to affecting DNA is calculated as being 12% v/v. A 10% v/v EC in water is predicted to be better than the optimum 12% v/v for formamide, with further improvements possible up to the optimum at about 50% v/v EC. All units are $MPa^{1/2}$. For definition of parameters see legend of Table S1.
