# Supplementary material for: Fast and Non-Toxic In Situ Hybridization without Blocking of Repetitive Sequences
Source: PLoS One. 2012 Jul 24;7(7):e40675. doi: 10.1371/journal.pone.0040675 (PMC3404051; doi:10.1371/journal.pone.0040675)
Supplement: Discussion S1 — HSP considerations in relation to the suggested base stacking mechanism. Please also see Table S1 and S2. (PDF) [file pone.0040675.s010.pdf]

Discussion S1

The aprotic solvents 2-pyrrolidone, sulfolane, δ-valerolactam, γ-butyrolactone, EC and propylene carbonate have similar Hansen solubility parameters (HSP) [18] characterized by high polar parameters (δ_P) with low or moderate hydrogen bonding parameters (δ_H) (Supporting Table S1). The useful function of these solvents is suggested to be a result of a lack of affinity for the DNA bases, which prevents interference of solvent molecules with the base pairing mechanism such that the strands rapidly re-anneal.

If the HSP of, for example, a 50% v/v mixture of EC and water is compared with the HSP of DNA, it can be seen that these values are close. This means that this mixture is a good solvent for DNA and therefore easily separates the strands sufficiently, even though neither of its ingredients has any individual affinity for the DNA bases. The solvents when mixed with water are ideal for denaturing the DNA because of the similarity in HSP, whereas formamide when mixed with water does not have the same ability even with the HSP similarity at its optimum amount of about 12% v/v (Supporting Table S2). The strands can more easily access and bind to their complementary strands when there is no interference from the solvent molecules. In addition, it can be seen from the HSP correlation for DNA (δ_H is lower than δ_P and δ_D) that it is not hydrogen bonding that controls the stability of the base pairing, but rather the insolubility of these bases in water that does not allow them to reside in the aqueous phase [18]. This supports the notion that the stability of the DNA helix is primarily due to hydrophobic stacking and not base pairing [29,30].
